# Supplementary material for: Clinically Expired Platelet Concentrates as a Source of Extracellular Vesicles for Targeted Anti-Cancer Drug Delivery
Source: Pharmaceutics. 2023 Mar 15;15(3):953. doi: 10.3390/pharmaceutics15030953 (PMC10056378; doi:10.3390/pharmaceutics15030953)
Supplement: Supplementary file 1 [file pharmaceutics-15-00953-s001.zip › pharmaceutics-2214821-supplementary.pdf]

pharmaceutics

Supplementary Materials: Clinically expired platelet concentrates as a source of extracellular vesicles for targeted anti-cancer drug delivery

Ana Meliciano [1,2,†], Daniela Salvador [1,2,†], Pedro Mendonça [3], Ana Filipa Louro [1,2], Margarida Serra [1,2,*]

[1] iBET, Instituto de Biologia Experimental e Tecnológica, Apartado 12, 2781-901 Oeiras, Portugal.
[2] ITQB-NOVA, Instituto de Tecnologia Química e Biológica António Xavier, Universidade Nova de Lisboa, Avenida da República, 2780-157 Oeiras, Portugal.
[3] IPST, Instituto Português do Sangue e da Transplantação, 1740-005 Lisboa, Portugal.

* Correspondence: mserra@ibet.pt
† These authors contributed equally to this work.

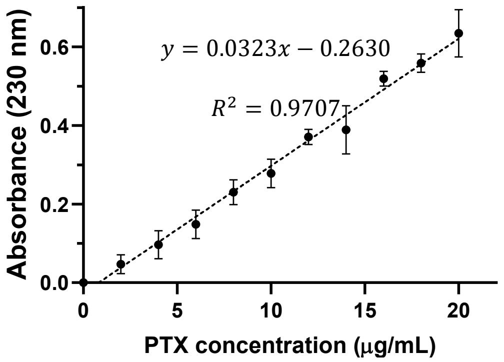

Figure S1. Paclitaxel calibration curve used to estimate PTX concentration into pEV. *n*=3, *n* represents independent experiments carried out inter-days.

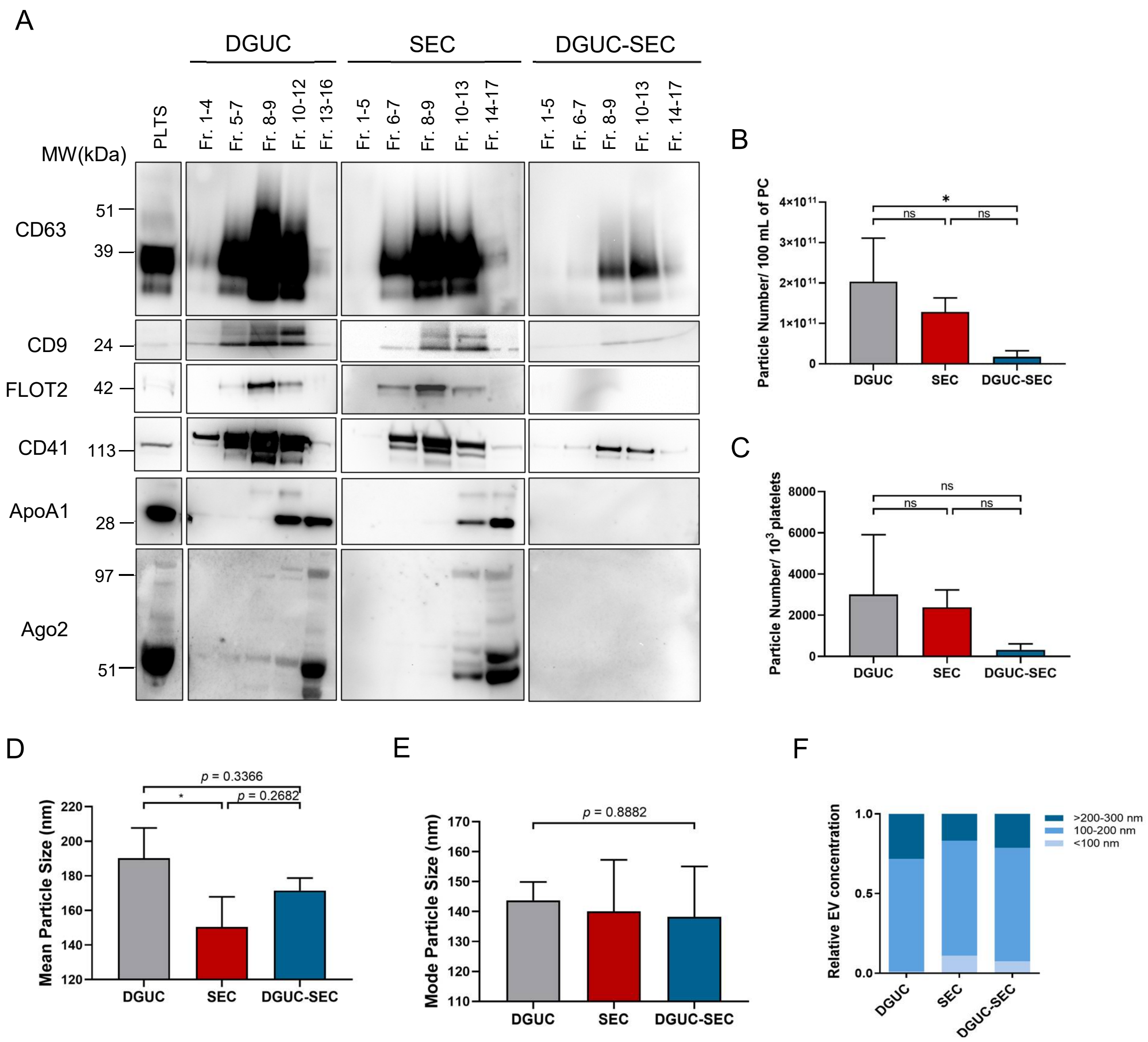

Figure S2. Characterization of pEV fractions isolated by DGUC, SEC, and DGUC-SEC in terms of purity, yield, and size distribution. **(A)** Western Blot analysis of specific EV markers (tetraspanins CD63 (glycosylated form) and CD9, and cytosolic protein flotillin-2), platelet-specific marker (CD41), and non-EV markers [apolipoprotein (ApoA1) and argonaute 2 (Ago2)] in pooled fractions and platelet lysate (PLTS). Molecular weight markers (MW, KDa) are indicated. **(B)** pEV-enriched fractions (Fr.8-9) were analyzed using nanoparticle tracking analysis. Particle number was normalized to platelet concentrate (PC) volume (100 mL) and **(C)** platelet count (10^3 platelets); (*n=3*). **(D)** Mean and **(E)** mode particle size (nm) of pEV samples; (*n=3*). **(F)** Relative pEV distribution ranged into three different size classes: < 100 nm, 100-200 nm and >200-300 nm; (*n*=3). *n* represents biologically independent replicates. Data are represented as mean ± S.D. *p < 0.05, n.s. not significant, determined by one-way ANOVA followed by Tukey's multiple comparison test.

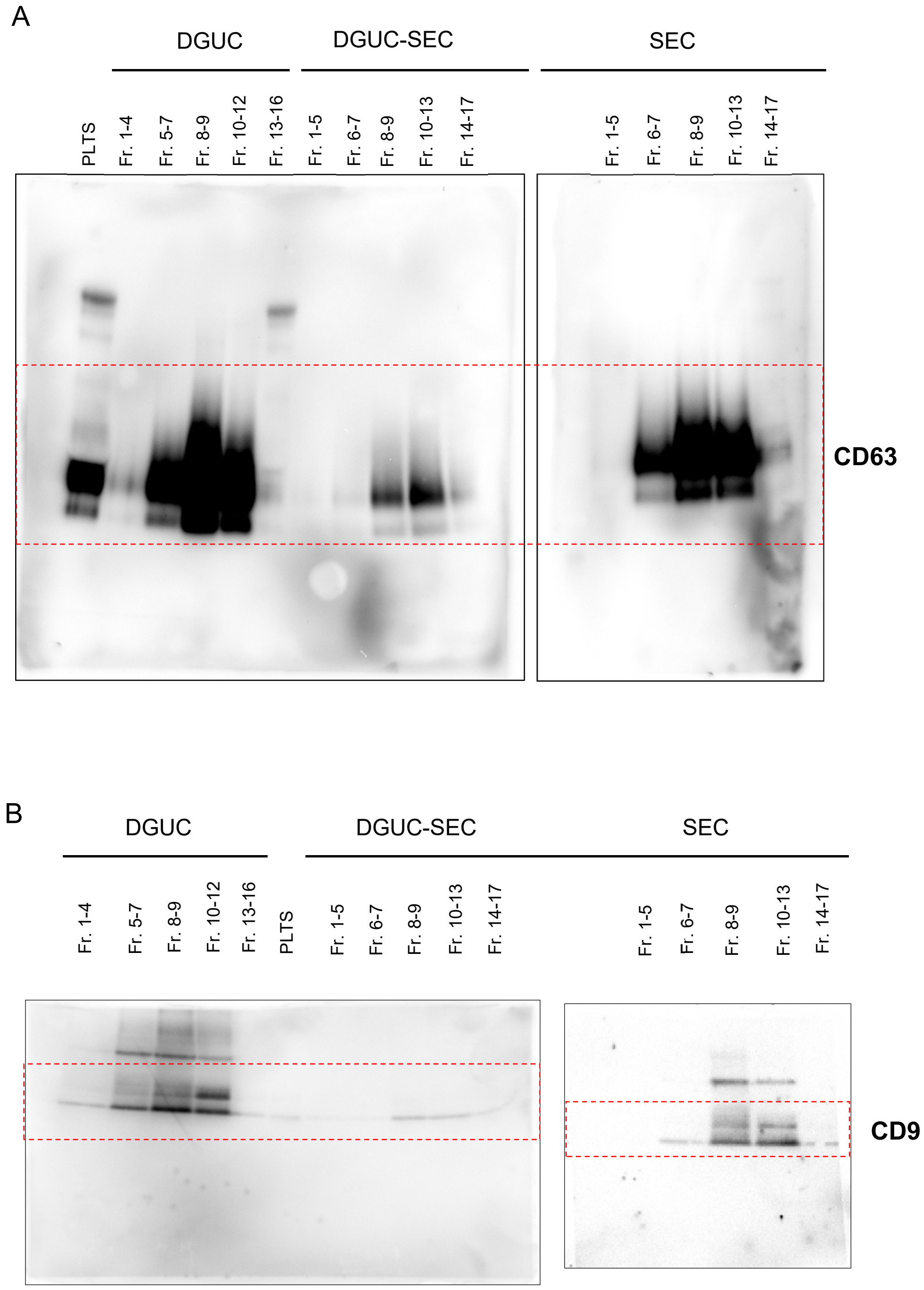
A
DGUC
DGUC-SEC
SEC
PLTS
Fr. 1-4
Fr. 5-7
Fr. 8-9
Fr. 10-12
Fr. 13-16
Fr. 1-5
Fr. 6-7
Fr. 8-9
Fr. 10-13
Fr. 14-17
CD63
B
CD9

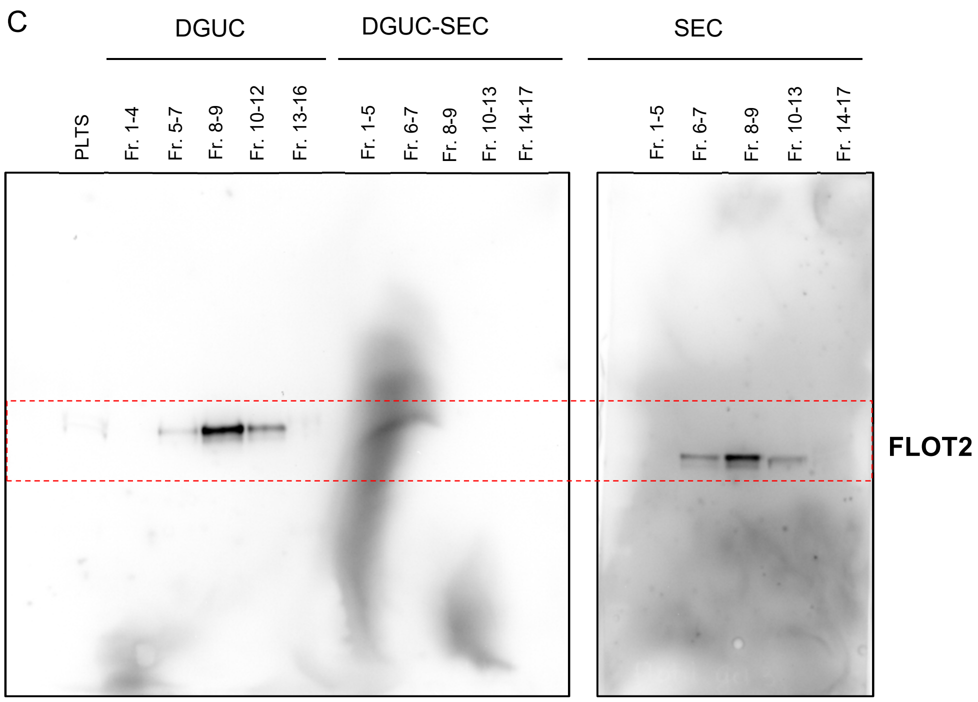
C
DGUC
DGUC-SEC
SEC
PLTS
Fr. 1-4
Fr. 5-7
Fr. 8-9
Fr. 10-12
Fr. 13-16
Fr. 1-5
Fr. 6-7
Fr. 8-9
Fr. 10-13
Fr. 14-17
Fr. 1-5
Fr. 6-7
Fr. 8-9
Fr. 10-13
Fr. 14-17
FLOT2

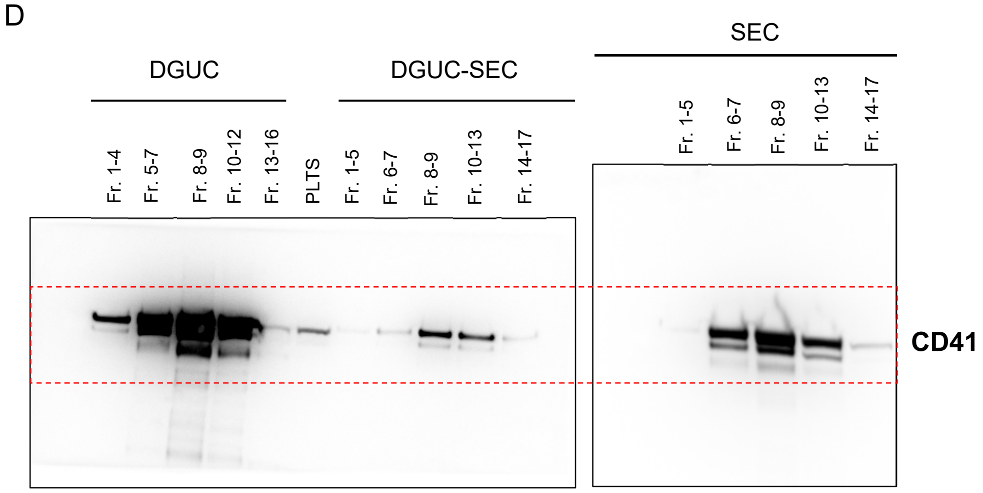
D
DGUC
DGUC-SEC
SEC
Fr. 1-4
Fr. 5-7
Fr. 8-9
Fr. 10-12
Fr. 13-16
PLTS
Fr. 1-5
Fr. 6-7
Fr. 8-9
Fr. 10-13
Fr. 14-17
Fr. 1-5
Fr. 6-7
Fr. 8-9
Fr. 10-13
Fr. 14-17
CD41

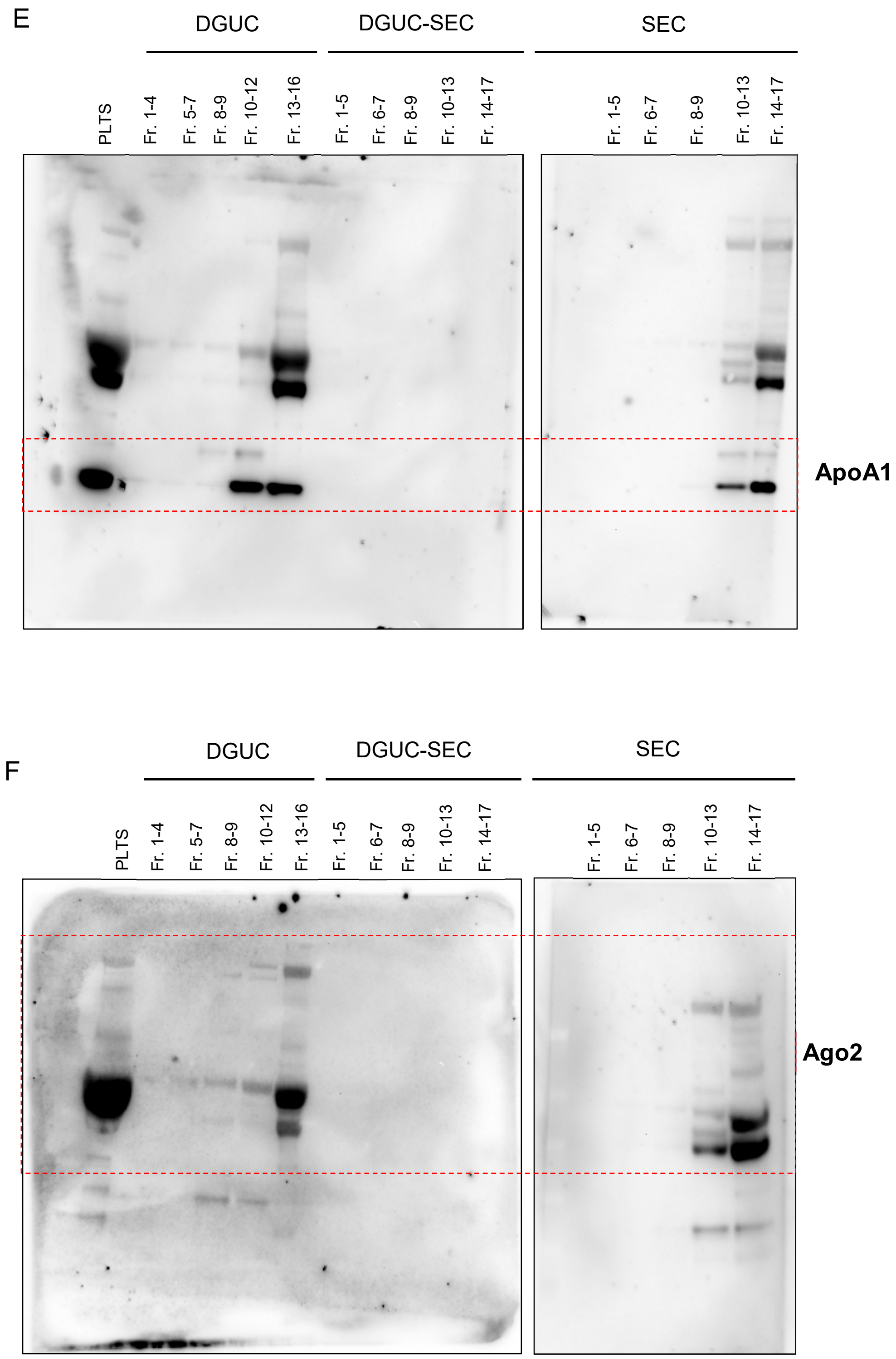

Figure S3. Full-length western blotting images for the results shown in main figures and supplementary Figure S2. Insets indicate cropped regions.

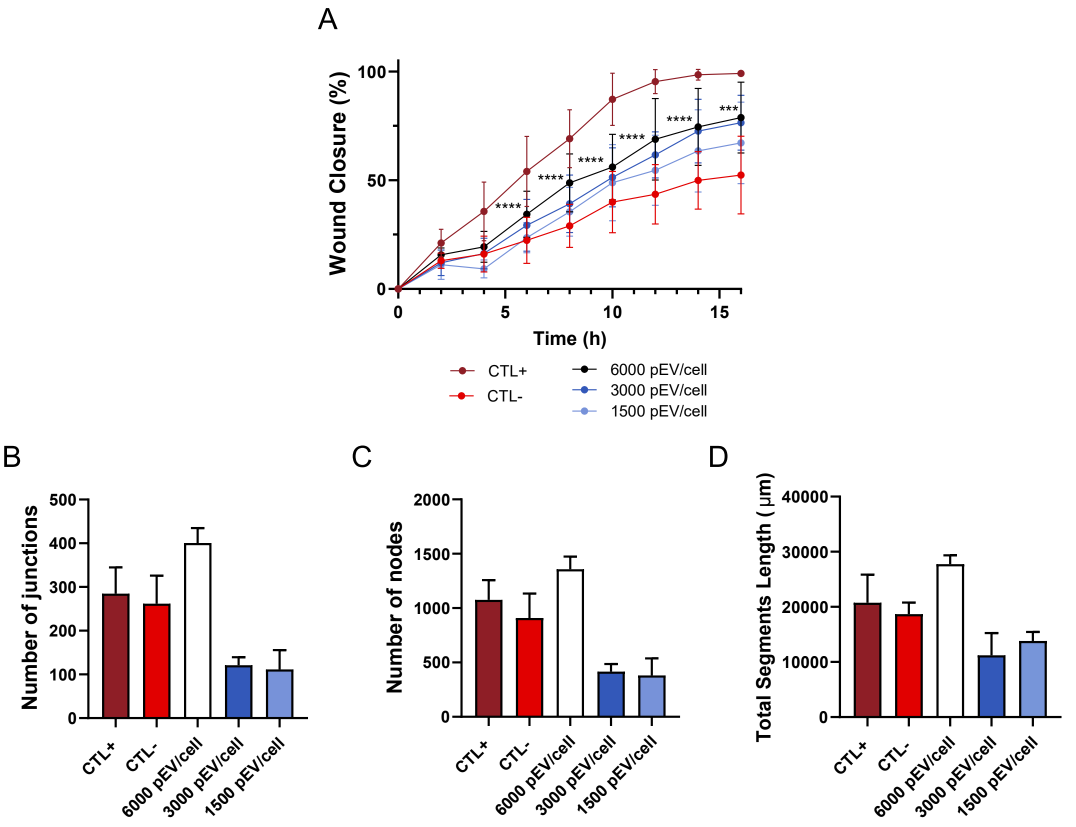

Figure S4. Effect of different pEV densities on HUVEC angiogenesis. **(A)** Quantitative analysis of the wound closure percentage throughout culture time. HUVEC were treated with 1500, 3000 and 6000 pEV/cell or with controls, CTL+ (cells cultured in supplemented media) and CTL- (8-9 fractions of DPBS gradient), for 16 h; (n=3). **(B)** Number of junctions, **(C)** nodes and **(D)** total segments length (µm) of HUVEC incubated with pEV or with controls, CTL+ and CTL-, for 8 h. Data are represented as mean ± S.D. One-way ANOVA followed by Tukey's multiple comparison test (6000 pEV/cell versus CTL-). ***$p < 0.001$, ****$p < 0.0001$.

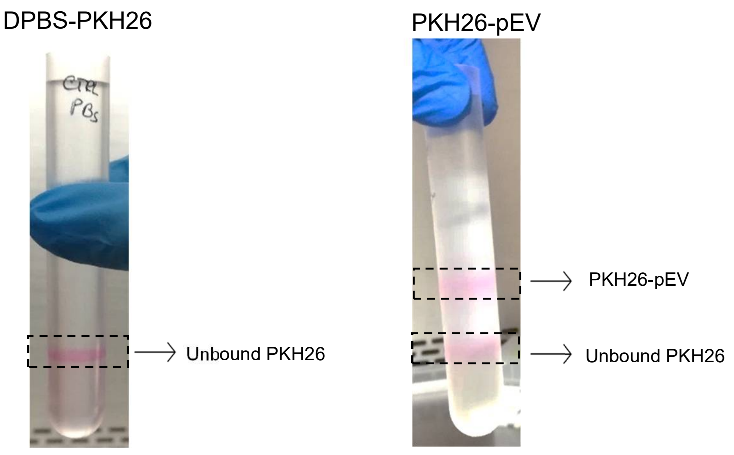

Figure S5. Separation of PKH26-pEV by DGUC. The presence of the two bands marked in the figure indicates a clear separation of PKH26-pEV (upper band) at the level of fractions enriched in pEV (fractions 8 and 9) from the unbound dye (lower band) located in denser fractions.

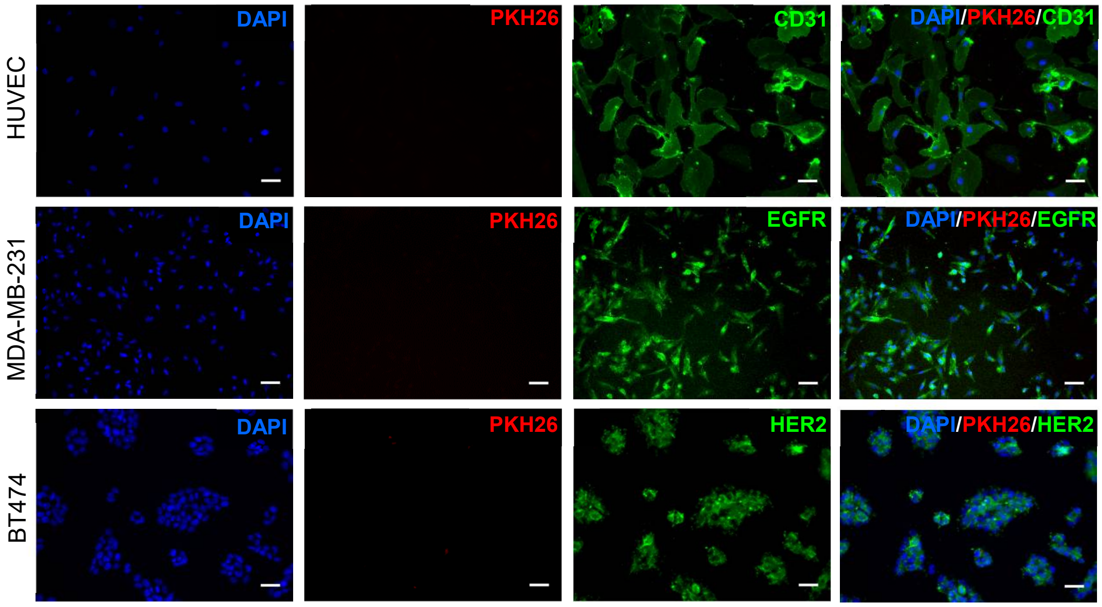

Figure S6. Cellular uptake of DPBS-PKH26 (negative control) by HUVEC, MDA-MB-231, and BT474 cells after 24 h of incubation. Representative immunofluorescence images of DPBS-PKH26 treatment. HUVEC, MDA-MB-231, and BT474 cells were stained for CD31, EGFR, and HER2 (green), respectively, and nuclei (DAPI, blue). Scale bars: 50 μm.

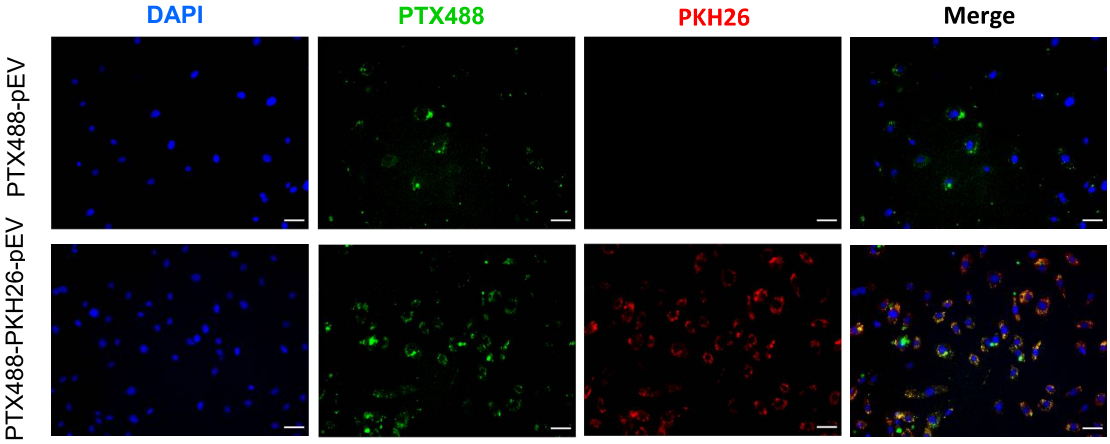

Figure S7. Intracellular distribution of PTX-loaded pEV in HUVEC cells after 24 h of incubation. HUVEC were treated with pEV loaded with tagged PTX (PTX488, green) and PTX488-pEV labeled with PKH26 (red) (PTX488-PKH26-pEV). Cell nuclei were stained with DAPI (blue), and cells were analyzed by fluorescence microscopy. Scale bars: 50 μm.
